# Effectiveness of Additional Preventive Measures for Pressure Injury Prevention in an Intensive Care Unit: A Retrospective Cohort Study

**DOI:** 10.3390/nursrep15070259

**Published:** 2025-07-16

**Authors:** Carolina Martín-Meana, José Manuel González-Darias, Carmen D. Chinea-Rodríguez, María del Cristo Robayna-Delgado, María del Carmen Arroyo-López, Ángeles Arias-Rodríguez, Alejandro Jiménez-Sosa, Patricia Fariña-Martín

**Affiliations:** 1 Intensive Care Unit, Hospital Universitario de Canarias, Carretera de Ofra s/n, 38320 La Laguna-Santa Cruz de Tenerife, Spain; josemanuelgonzalezdarias@gmail.com (J.M.G.-D.); cchirod@gobiernodecanarias.org (C.D.C.-R.); patrixia1293@gmail.com (P.F.-M.); 2 Department of Nursing, Faculty of Nursing, Campus de Ofra s/n, Universidad de La Laguna, 38200 San Cristóbal de La Laguna, Spain; crobayna@ull.edu.es (M.d.C.R.-D.); marroyo@ull.edu.es (M.d.C.A.-L.); 3 Department of Preventive Medicine and Public Health, Faculty of Health Sciences, Campus de Ofra s/n, Universidad de La Laguna, 38200 San Cristóbal de La Laguna, Spain; angarias@ull.edu.es; 4 Research Unit, Hospital Universitario de Canarias, Carretera de Ofra s/n, 38320 San Cristóbal de La Laguna, Spain; ajimsos@gobiernodecanarias.org

**Keywords:** pressure injury, moving average of the COMHON Index, additional measures, prevention, intensive care unit

## Abstract

**Background/Objectives**: Pressure injuries (PIs), a recognized indicator of care quality, have a higher incidence in intensive care units (ICUs). Our objective was to assess whether critically ill patients identified as “unprotected” (COMHON ≥ 11) developed pressure injuries despite additional preventive measures. **Methods:** A historical cohort study of an adult ICU was carried out. Of the 811 patients admitted in 2022, 400 were selected. All of them were subjected to the ICU’s PI Prevention Protocol, and those with a moving average of the COMHON Index ≥ 11 were given two additional measures: a multilayer dressing on the sacrum, and anti-equinus and heel-pressure-relieving boots. **Results:** A total of 36 patients presented with PIs (cumulative incidence of 9%). Significant differences were observed in their mean length of stay and in their disease severity score (APACHE-II). Most of the PIs were located on the sacrum, followed by the heel. Prior to the appearance of the PIs, a sacral dressing was applied to 100% of the patients, while anti-equinus and heel-pressure-relieving boots were only applied to 58.3%. Of the 36 patients with PIs, 52.8% had a PI on the sacrum and 22.2% on the heel. **Conclusions:** Focusing only on those who presented with PIs, we observed that the considered measures were not effective for preventing PIs in all the patients. Not all PIs are preventable, and individual risk factors should be considered. In the future, we will analyze the individual characteristics of these patients and verify whether the Prevention Protocol was followed, in order to determine how they could have been prevented or whether they were so-called unavoidable PIs.

## 1. Introduction

The presence of pressure injuries (PIs) in hospitalized patients is currently a recognized indicator of care quality. PIs, referred to as pressure injury until 2016 [1], are the result of ischemic necrosis at the level of the skin and subcutaneous tissues, generally caused by pressure exerted on a bony prominence [2], and are considered to be a mainly preventable adverse event of hospitalization [3]. Therefore, nursing professionals make constant efforts to reduce their incidence. Intensive care units (ICUs) are the perfect setting for the development of PIs, due to the characteristics of the patients who are admitted to them. Although no patient is completely free from the risk of developing a PI, those in ICUs are at higher risk due to neurological conditions, reduced mobility, altered body mass index (BMI), the use of vasopressors, nutritional deficiencies, etc. [4]. The occurrence of PIs in critically ill patients is associated with exposure to various factors inherent to their conditions, the care provided, the treatment, and/or inadequate prevention (i.e., the ineffectiveness or scarcity of preventive measures or the low priority given to their prevention). This results in the incidence rates of hospital-acquired PIs in ICUs being ten times higher compared to those in other hospital units [5,6].

PIs, although preventable in a high percentage of cases, continue to be a true epidemic, with significant human and economic costs. The treatment of PIs has significant direct, indirect, and intangible costs. Among the direct costs are the time spent by professionals, the cost of materials, increases in length of hospital stay, and the costs related to complications. The indirect costs include restrictions on the work activity of patients and caregivers, the costs of informal care or special equipment, years of life lost, legal costs, the use of social health resources, etc. The intangible costs include alterations to body image, stigmatization, pain, suffering, and impact on quality of life, among others [7]. In short, the treatment of PIs entails high costs for both patients and the healthcare system, in addition to the impact on the quality of life of both a patient and their family. This means that early intervention is essential for people at risk of developing PIs [8].

The prevention of PIs, as it is less costly than treatment, is the key intervention to protect patients from unnecessary harm. This requires an individualized care process aimed at reducing or controlling the risk factors [9].

However, the premise that some PIs are inevitable is increasingly recognized and accepted, and a consensus has been reached that there are certain intrinsic and non-modifiable factors—such as unstable hemodynamic status with repositioning, cardiovascular compromise affecting oxygenation and tissue perfusion, a shock state, and the use of invasive measures—where the implementation of certain strategies for the prevention of PIs is contraindicated [5,10,11,12]. Given this evidence, there is a need to develop and implement proactive, specific, and multifactorial preventive interventions aimed at critically ill patients, whose high susceptibility to the development of PIs is related to the complexity of their clinical situation and their limited mobility [13]. Following this line of reasoning, in an attempt to take a step further in addressing the challenges of protecting these critically ill patients from PIs, we applied a series of “Additional Preventive Measures” that were added to those included in the “PI Prevention Protocol” of our ICU. The additional measures chosen were a multilayer dressing on the sacrum, and anti-equinus and heel-pressure-relieving boots. These additional measures were justified as they are included in the “International Consensus on Preventive Interventions for PI According to Risk Level for Critical Patients” [14]. Therefore, the purpose of this research was to assess whether critically ill patients identified as “unprotected” (COMHON ≥ 11) developed PIs despite additional preventive measures.

The objective of this research was to determine, through a retrospective analysis, the occurrence of PIs in ICU patients identified as “unprotected” (moving average of the COMHON Index ≥ 11) after the implementation of two additional preventive measures.

## 2. Materials and Methods

The data were collected from digital medical records through the Critical Care Manager software program (version 8.2 Picis Inc., La Laguna, Canary Islands, Spain), where all of the care provided to the patients was recorded daily. The APACHE-II score was collected from a specific database in our ICU, which was managed exclusively by a responsible medical professional.

### 2.1. Design

This is a retrospective observational cohort study conducted during the year 2022. This study is reported in accordance with the STROBE reporting guidelines for observational research (cohort studies).

### 2.2. Study Setting and Sampling

#### Unit Description

This study was conducted in the adult ICU of a tertiary university hospital—specifically, at the Complejo Hospitalario Universitario de Canarias, part of the public network of the Servicio Canario de la Salud, on the island of Tenerife (Spain), which serves as a reference hospital for a population of 465,751 habitants, expanding to 1,650,228 habitants for the care of kidney transplant and cardiac surgery cases [15]. The ICU has 24 beds for the admission of critically ill patients due to medical and surgical conditions, as well as those recovering from cardiac surgery. The unit is staffed with medical personnel, including two nursing supervisors and five nursing teams, composed of 14 nurses and 7 auxiliary nursing technicians each, who work in 12 h shifts, with a nurse-to-patient ratio of approximately 1:1.71.

At the time of the study, the following care measures were standard in this ICU for the prevention of PIs: (1) risk assessment for PI at the time of admission, (2) risk reassessment every twenty-four hours, (3) moisture control, (4) pressure minimization, (5) oxygenation control through arterial blood gas analysis every three or four hours, and (6) enteral or parenteral nutrition with blood monitoring of electrolytes and proteins. Additionally, all admitted patients were subjected to the “PI Prevention Protocol” of our unit (Table 1).

For this study, two additional measures for the prevention of PIs were added, which were applied to those patients whose moving average of the COMHON Index was equal to or greater than 11 [16,17,18]. These additional measures consisted of the use of multilayer dressings in the sacral area and the placement of anti-equinus and heel-pressure-relieving boots. These measures were applied because the areas where the most PIs occurred in our ICU were the sacrum and the heel [17,18].

### 2.3. Population, Sample, and Sampling

The study population consisted of 811 patients over 18 years old who were admitted to the ICU during the year 2022. For the calculation of the sample size, the formula for estimating proportions in finite populations was used, with a confidence level of 95%, a margin of error of 0.05, and an expected proportion of 50%, which resulted in a minimum of 395 patients. Ultimately, 400 patients from the population who met the study’s inclusion criteria were included; their selection was carried out randomly, starting with the first patient who met the inclusion criteria, until the total sample size was reached.

### 2.4. Inclusion and Exclusion Criteria

Those patients who had the following recorded in their medical history were included: their moving average of the COMHON Index, the use or non-use of the additional measures proposed in this study (i.e., a multilayer dressing on the sacrum and anti-equinus and heel-pressure-relieving boots), and the presence or absence of PIs.

Those patients who did not have some of the aspects considered as inclusion criteria recorded in their medical history were excluded.

### 2.5. Study Subjects

The subjects were patients over 18 years old who were admitted to the ICU of the Complejo Hospitalario Universitario de Canarias during the year 2022. Patients with various pathologies and those who had undergone cardiac surgery were admitted to this unit. The patients were grouped into three categories, according to their diagnosis: medical, surgical, and cardiac surgery.

The data used to calculate the incidence of PIs were collected from the first day of ICU admission until discharge, death, or a maximum ICU stay of 35 days.

### 2.6. Instruments for Data Collection

For the assessment of the risk of a PI, the specific COMHON Index scale for intensive care was used [19]. For the calculation of the level of protection against the occurrence of a PI, the technique of a continuously updated “three-day moving average” was used, which is the arithmetic mean of the risk score values obtained with the COMHON Index [18,20]. To measure the severity of the disease, the APACHE-II score (an acronym in English for “Acute Physiology And Chronic Health Evaluation II”) was used, which is a classification system for the severity or seriousness of diseases [21]. This was applied within the first 24 h after a patient’s admission to the ICU, and yielded an integer value between 0 and 67. A higher score corresponds to more severe disease and a greater risk of death, influencing the predisposition of critically ill patients to developing PIs [18,20,21,22,23]. The source of the information used in this study for the data collection was the records from the Critical Care Manager TM v.8.2 system (Picis Inc., La Laguna, Canary Islands, Spain), where the nurse responsible for the patient’s care recorded the variables included in this study, except for the APACHE-II score, which was recorded by the ICU doctor assigned to this task at the time of the patient’s admission and noted in a specific database where these data were collected (it was recorded only at ICU admission, which limits its representativeness over time).

### 2.7. Description of the Variables

#### 2.7.1. Dependent Variable

The dependent variable was the documented diagnosis of at least one pressure injury or absence of PIs.

#### 2.7.2. Independent Variables

- Moving average of the COMHON Index: A minimum value of 5, and a maximum of 20. Values equal to or greater than 11 indicate that a patient is not protected against the occurrence of PIs.
- Additional measures:
  - The application of multilayer dressing on the sacrum.
  - The placement of anti-equinus and heel-pressure-relieving boots.
- The other independent variables considered in this study included the following: age (in years), sex (male or female), days of stay in the ICU, reason for ICU admission (medical diagnosis, surgical diagnosis, or cardiac surgery diagnosis), performance of postural changes prior to the appearance of a PI, anatomical location of PIs (sacrum, heel, malleolus, buttock, forehead, cheekbone, chin, acromion, breasts, abdomen, genitals, knees, tibial plateau, dorsum of the feet, toes, etc.), number of PIs per patient, body mass index (BMI) [24,25,26], and APACHE-II score.

### 2.8. Statistical Analysis

Categorical variables are expressed as frequencies and percentages.

Quantitative and ordinal variables are expressed as means and standard deviations or medians and interquartile ranges (25–75), depending on whether data followed normal or asymmetric distribution, respectively. Proportional comparisons were conducted using chi-squared tests or Fisher’s exact tests, as appropriate.

Group comparisons of quantitative and ordinal variables were performed using Mann–Whitney test. Values of *p* < 0.05 were considered significant.

Data analyses were conducted using SPSS 25.0 statistical package (IBM, 2017, Armonk, NY, USA).

### 2.9. Ethical Aspects

This study was conducted in accordance with the guidelines of the Declaration of Helsinki and was approved by the Clinical Research Ethics Committee of the University Hospital Complex of the Canary Islands for the Province of Santa Cruz de Tenerife, with the reference code CHUC_2021_66 (Pro LPP_2).

## 3. Results

Of the 400 patients in our sample, 36 developed PIs, corresponding to a 9% cumulative incidence. The average stay in the ICU was 8.61 days, and the patients who developed PIs were admitted for a median of 15 days (IQR: 8–26 days). The sample had a mean age of 64.4 years, and 64.75% were men. In Table 2, the clinical and demographic characteristics of the cohort are presented based on the presence or absence of PIs. No statistically significant differences (*p* < 0.05) were found when comparing the patients with and without PIs in terms of their age, sex, BMI, and diagnosis upon ICU admission. Significant differences were found when comparing the two groups in terms of their mean duration of stay in the ICU (in days) and their APACHE-II scores.

Among the patients who presented with PIs, the average time of appearance of the first PI was 7.31 (±6.4) days, and the mean moving average of the COMHON Index on the first day of the appearance of the PI was 13.44 (SD 3.39).

Of the 36 patients who presented with PIs, some had more than one PI (see Table 3), resulting in a total of 49 PIs.

**Figure 1:**
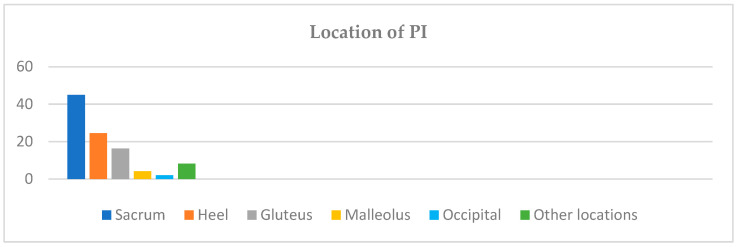
PI distribution by anatomical location.

The majority of the PIs were located on the sacrum (44.9%), followed by the heels (24.5%), gluteus (16.3%), malleolus (4.1%), occipital area (2%), and other locations not specified in the registry (8.2%) (see Figure 1).

Table 4 shows that 100% of the patients with PIs were given an additional preventive measure of a multilayer dressing on the sacrum, while anti-equinus and heel-pressure-relieving boots were provided to only 58.3% of the patients with PIs, on a preventive basis. It should be noted that, during this period, there were problems with the supply of this material in our hospital, which could justify these data.

These data show that despite having applied a multilayer dressing on the sacrum as an additional preventive measure for all the patients, 22 presented with a sacral PI. Regarding the additional measure of anti-equinus and heel-pressure-relieving boots, which were only provided to 21 out of the 36 patients who had PIs, Table 5 shows the distribution of the PIs on the heels and malleolus in the patients wearing or not wearing such boots.

The results obtained show that even after applying these additional measures to the patients with a moving average of the COMHON index ≥ 11, which indicates that a patient is “not protected” against the appearance of PIs by the usual protective measures, the patients developed PIs. This led us to analyze whether the 36 patients who presented with PIs underwent postural changes every 2–3 h, as required by our ICU’s PI Prevention Protocol. We found that 13 (26.1%) patients did not undergo postural changes in the hours prior to the onset of PIs.

## 4. Discussion

At present, the incidence and prevalence of PIs in seriously ill patients admitted to ICUs remain high, despite a great deal of knowledge about the appropriate prevention strategies [27,28]. The results obtained in this study show a cumulative PI incidence of 9%. The calculation of this rate did not exclude the patients who presented with PIs in the first 24 h after admission to the ICU because, as this was a retrospective study, the records did not differentiate whether the patients had been admitted with PIs or whether they developed after admission to the ICU. If we had discounted them, our cumulative PI incidence rate would have been 7.25%—an incidence rate more consistent with our latest studies [17,18,20,29]. Coinciding with the results of other studies, when analyzing the age, sex, and reason for admission, we found no significant differences between the patients with and without PIs. With respect to the BMI, we did not observe any significant differences, unlike other studies [25]; although it should be noted that the patients in our study had an average BMI corresponding to overweight values. On the other hand, we observed a significant relationship between the time spent in the ICU and the incidence of PIs, coinciding with the results of other studies [16,17,18,20,29,30]. However, we observed a relationship between the APACHE-II score and the presence of PIs. This result is consistent with other studies that have established a relationship between the APACHE-II score and the occurrence of PIs [31,32,33], but it is not consistent with previous studies conducted in our ICU that have observed that the severity of the disease—determined by the APACHE-II score—is not an independent risk factor for developing a PI, since it is calculated only during the first 24 h after admission. Therefore, it is not representative of the severity of disease during a several-day ICU stay; in addition, it could be attributed to the influence of the nursing care received by our patients [18,20,29]. The median APACHE-II score of the patients with PIs was 18.50, with an IQR of 12–26.50. In the literature, the APACHE-II score is taken as a predictor of mortality. A score between 15 and 19 is associated with 25% mortality, values between 20 and 24 with 40% mortality, and values of 25–29 with 55% mortality [21,22].

The value of the moving average of the COMHON Index corresponding to the day on which the patients presented with their first PI was 13.44, in accordance with other studies where a score ≥ 11 [18,20] was identified as the optimal cut-off point and was associated with a risk of presenting with PIs. The most frequent location of the PIs was the sacrum, followed by the heels— coinciding with the findings of other studies [34,35,36,37,38,39,40] on patients admitted to ICUs. The majority of the patients who developed PIs had only one, and almost one-third of them had two or more PIs.

As for performing postural changes every 2–3 h—a measure included in our ICU’s PI Prevention Protocol—just over one-quarter of the patients with PIs were not subjected to this. This could have been justified by the patient’s clinical situation at that time, e.g., the presence of hemodynamic and respiratory involvement [41,42,43].

Regarding additional measures, a multilayer dressing on the sacrum was applied to all the patients preventatively, but this did not prevent nearly half of the PIs appeared located in that area. The anti-equinus and heel-pressure-relieving boots (which could not be applied to all the patients due to problems with the supply of this material during the study) were also shown to not be an effective measure in all the patients, since most of the patients who had PIs on their heels and all of those who had PIs on the malleolus used them. This, according to Gefen et al. [44], could be related to the concept of individual susceptibility to PIs, which depends on integrated body system functions and is dynamic and extremely difficult to predict in seriously ill patients. We should also consider the relationship between PIs and the unit-based data [45]; additionally, targeted educational programs for ICU nurses should be developed to enhance their knowledge and skills in PI management [46].

## 5. Limitations

The retrospective nature of the study limits the ability to establish causal relationships. The reason that only 58.3% of the patients with PIs wore anti-equinus and heel-pressure-relieving boots prior to the onset of PIs was mainly due to a discontinuity in the supply of this material. Another potential limitation of this study may be the interactions of other variables not considered here (e.g., comorbidities, progressive clinical severity), which may have influenced the results.

## 6. Conclusions

Of the 400 patients included in this study, only 36 patients (9%) developed pressure injuries. Most of the PIs were located on the sacrum, followed by the heels—which were the target of additional preventive measures. All the patients with PIs on the sacrum had a multilayer dressing on the sacrum, and most of the patients with PIs on the heels had anti-equinus and pressure-relieving boots. If we focus only on the patients who had PIs despite the additional measures applied, we can conclude that these measures were not fully effective at preventing PIs in these areas in all the patients. The individual characteristics of these patients should be analyzed, and it should be determined whether all of the measures included in the Prevention Protocol were carried out, in order to assess the adherence to the protocol and identify whether the injuries could have been prevented or were potentially unavoidable.

We propose that upcoming studies within our research line focus on analyzing the characteristics of critically ill patients with PIs who are subject to severe life-threatening conditions.

## Figures and Tables

**Table 1:** PI Prevention Protocol applied to the study patients.

PI Prevention Protocol of the ICU of the Complejo Hospitalario Universitario de Canarias
Incorporate a dynamic mattress on the bed.
Monitor the skin thoroughly during bathing and when performing position changes.
Perform proper drying of the skin, without friction and with the subsequent application of hydration.
Avoid excessive use of clothing under patients (tucked-in sheets and folded sheets).
Apply hyperoxygenated fatty acids to at-risk areas.
Apply barrier products to areas exposed to moisture (e.g., breast folds, abdominal folds) or exposed to urinary and/or fecal incontinence.
Make postural changes every 2–3 h.
Use special surfaces for pressure management.
Keep the heels pressure-free and use multilayer dressings.
In neurocritical patients, closely monitor the occipital area and use offloading devices, such as multilayer dressings or viscoelastic surfaces.
Measure the risk of PIs using the COMHON Index scale [11,12,16].
Calculate the moving average [17] of the COMHON Index to determine the level of protection that a patient has against PIs.
**The specific protocol for patients in the prone position:**
Apply multilayer dressings or viscoelastic surfaces to the at-risk areas (e.g., forehead, cheeks, chin, acromion, breasts, abdomen, genitals, knees, tibial plateau, dorsum of the feet, and toes).
Perform mobilizations of the hips and the upper and lower limbs to relieve local pressure.
Strictly monitor the areas where monitoring and/or therapeutic devices are placed, to prevent the occurrence of iatrogenic injuries.

**Table 2:** Clinical and demographic characteristics of the patients.

Characteristics	NO PI n = 364	PI n = 36	*p*-Value
**Median age, years (IQR)**	66 (56.25–75)	66.50 (62–75.50)	0.41
**Sex:**			0.32
Men, n (%)	233 (64)	26 (72.2)	
Women, n (%)	131 (36)	10 (27.8)	
**Average length of stay in ICU, days (IQR)**	2.50 (1–6)	15 (8–26)	*p* < 0.001
**Body mass index (BMI), (IQR)**	27.68 (24.68–31.01)	29.71 (24.60–32.47)	0.35
**APACHE-II score (IQR)**	12 (7–17)	18.50 (12–26.50)	0.002
**Diagnosis, n (%):**			0.42
Medical	**152 (41.8)**	**18 (50)**	
Surgical	**105 (28.8)**	**11 (30.6)**	
Cardiac surgery	**107 (29.4)**	**7 (19.4)**	

PI (pressure injury), APACHE-II (Acute Physiology and Chronic Health Evaluation). The data are expressed in frequencies (%) or medians (IQR: interquartile range, P25–P75).

**Table 3:** Number of PIs per patient.

Number of PIs	Patients with PIs n = 36 (%)
One	26 (72.2)
Two	7 (19.4)
Three	3 (8.3)

Data are expressed as frequencies (%).

**Table 4:** Application of additional measures in patients with PIs.

Additional Measures	Patients with PIs n (%)
Multilayer sacral dressing	36 (100)
Anti-equinus and heel-pressure-relieving boots	21 (58.3)

Data are expressed as frequencies (%).

**Table 5:** Presence of PIs on the heels and malleolus related to the application of anti-equinus and heel-pressure-relieving boots.

Anti-Equinus and Heel-Pressure-Relieving Boots	Heel PIs n = 12	Malleolus PIs n = 2
Yes	5 (41, 6)	2 (100)
No	7 (58, 3)	0 (0)

Data are expressed as frequencies (%).

## Data Availability

The data used in this research are confidential and are stored in a coded and anonymized database, managed by the research group in accordance with Spanish regulations. However, the raw data may be shared with researchers who contact the corresponding author, provided they submit a reasoned and justified request.

## Public Involvement Statement

There was no public participation in any aspect of this research.

## Guidelines and Standards Statement

This manuscript was written in accordance with the Statement on Strengthening Reporting of Observational Studies in Epidemiology (STROBE): Guidelines for Reporting Observational Research (Cohort Studies).

## Abbreviations

The following abbreviations are used in this manuscript:

AI	Artificial Intelligence
APACHE-II	Acute Physiology And Chronic Health Evaluation Score II
COMHON	Consciousness Level–Mobility–Hemodynamics–Oxygenation–Nutrition
IBM	International Business Machines
BMI	Body Mass Index
PI	Pressure Injury
NY	New York
